# Factors associated with depression symptoms in women after breast cancer

**DOI:** 10.11606/S1518-8787.2019053000786

**Published:** 2019-03-27

**Authors:** Leonessa Boing, Gustavo Soares Pereira, Camila da Cruz Ramos de Araújo, Fabiana Flores Sperandio, Monique da Silva Gevaerd Loch, Anke Bergmann, Adriano Ferreti Borgatto, Adriana Coutinho de Azevedo Guimarães

**Affiliations:** I Universidade do Estado de Santa Catarina. Programa de Pós-Graduação em Ciências do Movimento Humano. Florianópolis, SC, Brasil; II Universidade do Estado de Santa Catarina. Departamento de Educação Física. Área de Ciências do Movimento Humano. Florianópolis, SC, Brasil; III Universidade do Estado de Santa Catarina. Departamento de Fisioterapia. Florianópolis, SC, Brasil; IV Universidade do Estado de Santa Catarina. Departamento de Ciências de Saúde. Florianópolis, SC, Brasil; V Instituto Nacional do Câncer. Rio de Janeiro, RJ, Brasil; VI Universidade Federal de Santa Catarina. Departamento de Informática e Estatística. Florianópolis, SC, Brasil; VII Universidade do Estado de Santa Catarina. Departamento de Educação Física. Florianópolis, SC, Brasil

**Keywords:** Women, Depression, Breast Neoplasms, psychology, Body Image, Socioeconomic Factors, Cross-Sectional Studies

## Abstract

**OBJECTIVE:**

To analyze the factors associated with the presence of depression symptoms in women after breast cancer.

**METHODS:**

Cross-sectional study with 181 women with breast cancer, aged 57.0 years (SD = 9.5), who were undergoing treatment or after treatment in the Oncology Research Center in Florianópolis, state of Santa Catarina, Brazil. The questionnaire comprised items addressing general and health information, economic level, anthropometric measures, depression symptoms (Beck Depression Inventory), self-esteem (Rosenberg Self-Esteem Scale), and body image (Body Image After Breast Cancer Questionnaire). Descriptive and inferential statistical analysis were performed by chi-square and Fisher's exact tests to verify association, Mann-Whitney *U* test to compare the groups and Poisson regression to identify the prevalence ratio of the factors associated with presence of depression symptoms (p < 0.05).

**RESULTS:**

We found an association between the presence of depression symptoms and the group of younger women (aged 40–60 years), those who had another disease besides cancer, those who had mastectomy surgery, those who suffered from lymphedema, and those who presented low–medium self-esteem. Less educated women presented more depressive symptoms, as did women with worse body image on the subscales of limitations, transparency, and arm concerns.

**CONCLUSIONS:**

Age, educational attainment, diagnosis of other diseases, type of surgery, lymphedema, self-esteem, and body image were factors associated with the presence of depression symptoms in Brazilian women after breast cancer. Health professionals should be aware of these relationships and try to detect depression symptoms earlier and improve the care they provide to these women.

## INTRODUCTION

The perception of female appearance has evolved over the years. It is influenced by the media, which encourages women to achieve an ideal body^1^. In this sense, breast size is related with femininity and the ideal definition of beauty^2^. Consequently, when women are diagnosed with breast cancer (BC), and their breasts undergo a physical transformation, it likely compromises their sexual identity, interpersonal relationships, and sense of self^3^. Therefore, BC and its treatment could influence body image and female identity in various ways, as well as decrease women's self-esteem^1^ ^–^ ^3^. Consequently, this situation could bring upon depressive symptoms because it evokes feelings of inferiority and fear of rejection from one's partner, children, or friends^4^.

Depression is one of the most common psychological consequences of BC^4^ ^,^ ^5^. It has been diagnosed in one out of five BC women^6^ and negatively affects women's quality of life^7^. As depression decreases immunity and chances of survival^5^, interventions that improve overall health are relevant for BC treatment. Studies that address aspects of self-esteem and body image and their relationship with BC are highly relevant because it is considered a public health problem^8^. BC is the most recurrent cancer among women^9^ and has a high mortality rate and serious psychological repercussions. In this regard, this study analyzed the factors associated with depression symptoms in Brazilian women who had been diagnosed with BC.

## METHODS

### Study Design

This study has an analytic, observational, and cross-sectional design.

### Participants

One-hundred and eighty-one women previously diagnosed with BC, undergoing treatment or after treatment at the Oncological Research Center (CEPON) in the city of Florianópolis, Santa Catarina, Brazil, voluntarily participated. The sample size was calculated using the software G*Power 3.1.9.2^10^. Considering a significance level of 5%, power of 80%, a prevalence ratio effect size of 1.62, and the Poisson Regression test, 182 participants were required.

Inclusion criteria were: (a) being aged between 40 and 80 years, considering that having more than 40 years old increased the chance of a BC diagnosis; (b) being in any phase of adjuvant or neoadjuvant treatment at the hospital, or being medically monitored after finished the clinical treatment. Exclusion criteria comprised: (a) being classified as illiterate, since the subject could have difficulty of understanding the questions; and (b) displaying signs of clinical stage IV of cancer (presence of metastasis), to avoid treatment and prognostic bias. From the total of 281 women interviewed, 100 were not eligible (54 because presented stage IV of BC and 46 were younger than 40 years old), resulting in 181 women included in the final sample.

The study was approved by the Ethics Committee of the institution (Protocol 688,548) and by the Research Ethics Committee of CEPON (Protocol 818,174).

### Data Collection

Data was collected from October 2014 to July 2015 using a structured questionnaire applied to participants in individual interviews by three female trained researchers. All participants signed an informed consent form.

All contacts were made inside the research center in the following wards: chemotherapy, radiotherapy, physical therapy, and in the waiting room of the doctors' offices and examination rooms. The interviews lasted approximately 30 minutes.

### Variables

The main instrument, a questionnaire, was constructed specifically for this study and included participants' general characteristics, independent variables (self-esteem and body image) and the primary outcome variable (depression symptoms).

### General Characteristics

To describe the study population, we investigated the general characteristics (i.e., age, marital status, educational attainment, economic level, present job, weight status) and disease characteristics (i.e., treatment stage, type of surgery, breast reconstruction, presence of other diseases, presence of lymphedema, physical therapy treatment). Economic level was investigated according to the Brazilian Institute of Geography and Statistics (IBGE) criteria^11^, relativized by minimum wage: class A with family income corresponding to more than 20 minimum salaries; class B with 10 to 20 minimum salaries; class C with four to 10 minimum salary; class D with two to four minimum salaries; and class E with up to two minimum salaries. For this study, the minimum salary of 2014 of R$724,00 was considered and the participants were classified into high level (A+B), middle level (C), and low level (D+E), for statistical purposes. The anthropometric measures (weight and height) were self-reported. Body mass index (BMI) was used to classify nutritional status according to the World Health Organization criteria^12^.

### Self-Esteem

The Rosenberg Self-Esteem Scale (1979) was internationally validated for populations with cancer^13^ and for the Brazilian population^14^. It was used in other studies addressing patients with BC^3^ ^,^ ^9^ ^,^ ^15^. The questionnaire was formed by 10 affirmations involving self-esteem and self-acceptance that resulted in global self-esteem. Total scores ranged from 10–40. Participants' self-esteem was sorted into: satisfactory or high (≤ 31 points), average (21–30 points), and unsatisfactory (≤ 20 points).

### Body Image

The Body Image After BC questionnaire was developed by Baxter et al.^16^ and translated, validated, and culturally adapted in Brazil by Gonçalves et al.^17^ It is a specific instrument used to verify the perception of body image after a BC diagnosis. The questionnaire is composed of 44 questions divided into six scales: vulnerability, body stigma, limitations, body concerns, transparency, and arm concerns. Data interpretation depended on the magnitude of the sum of the scores; the higher the score, the higher the relation to body image^17^.

### Depression Symptoms

The Beck Depression Inventory (BDI) developed by Beck et al.^18^ is a self-report questionnaire with 21 multiple-choice questions that characterize depression symptoms (i.e., lack of hope, anger, cognition, guilt, and punishing feelings) and physical symptoms (i.e., fatigue, weight loss, and diminished sexual interest). Cunha^19^ validated the BDI in Brazil. BDI has been used in studies analyzing women with BC^4^ ^,^ ^15^. Individual scores are summed and the scale has a maximum value of 63 points. Higher scores indicated a higher degree of depression and a value of zero indicated the absence of depression symptoms.

In this study, categorization was performed according to the Cognitive Therapy Center standards for cancer patients: scores from 0–10 indicated without or minimal depression; 11–18, mild depression; 19–29, moderate depression; and 30–63, severe depression^20^. For statistical procedures, the groups were categorized as absence of depression symptoms (scores from 0–10) and presence of depression symptoms (scores ≥ 11). The term “depression” was not used given that it is typically related to patients with a concomitant clinical diagnosis^20^.

### Statistical Analysis

The chi-squared test and Fisher's exact test was used to compare the general characteristics of the disease and self-esteem between the groups with and without depression symptoms, and the Mann-Whitney *U* test was used to compare the groups in body image scores because the data failed to present normality using the Kolmogorov-Smirnov test (p > 0.05).

To estimate the brute and adjusted prevalence ratio with their respective confidence intervals, the Poisson regression with robust variance was calculated. The dependent variable was the presence of depression symptoms. In the adjusted analysis, the conceptual model presented in Figure was used for all variables: general characteristics in the first block, disease characteristics in the second block, and self-esteem and body image in the last block. The limit value to maintain the variable in the adjusted analysis was p ≤ 0.20, which was also presented in the regression table for each block.

**Figure:**
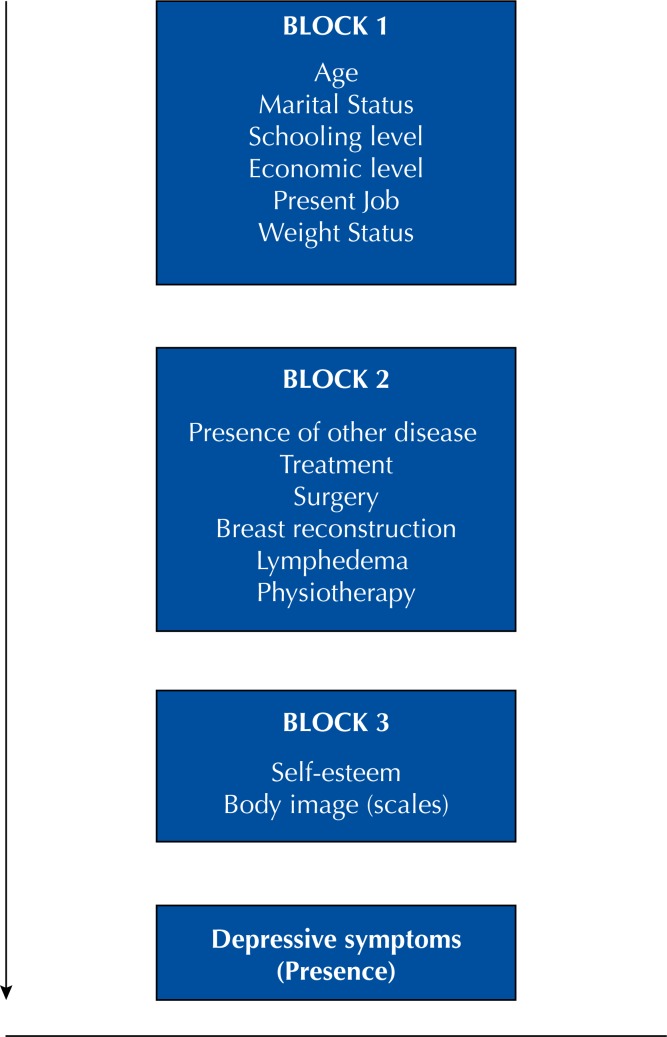
Conceptual model used in Poisson Regression. Florianópolis, Santa Catarina, Brazil, 2017.

## RESULTS

The prevalence of depression symptoms in women after a BC diagnosis in this study was 49.2% (95%CI 41.8–56.5); 56.3% (95%CI 45.6–66.6) presented mild symptoms; 30.3% (95%CI 20.6–40.0) presented moderate symptoms; and 13.5% (95%CI 6.2–20.7) presented severe symptoms (data not shown).

Women aged between 40–60 years presented more depressive symptoms than did women aged 61–80 years. Less educated women had more depressive symptoms than their higher-educated counterparts. Most of the sample had a partner and was overweight, unemployed or retired, and from a low socioeconomic level (D+E) (Table 1).

**Table 1:** Women's personal characteristics according to the two groups of depression symptoms. Oncological Research Center (CEPON). Florianópolis, state of Santa Catarina, Brazil, 2014/2015. (n = 181)

Variable		Total	Absence of depression symptoms	Presence of depression symptoms	p
		% (95%CI)	%	%	
Age (years old)					**0.042** a
	40–60	65.7 (59–73)	58.7	73.0	
	61–80	34.4 (27–41)	41.3	27.0	
Educational attainment					**0.006** a
	Basic school	53.6 (46–60)	43.5	64.0	
	High school and undergraduate degree	46.4 (39–53)	56.5	36.0	
Present job					0.514^a^
	Home	26.5 (20–33)	25.0	28.1	
	Unemployed/Retired/Expertise	58.0 (51–65)	56.5	59.6	
	One or more jobs	15.5 (10–21)	18.5	12.4	
Marital status					0.576^a^
	With partner	57.5 (50–65)	55.4	59.6	
	Without partner	42.5 (35–50)	44.6	40.4	
Economic level					0.661^b^
	Low level (D+E)	82.3 (77–88)	80.4	84.3	
	Medium level (C)	13.8 (9–19)	16.3	11.2	
	High level (A+B)	3.9 (1–7)	3.3	4.5	
Weight status^c^					0.681^a^
	Overweight	72.5 (66–79)	71.1	73.9	
	Healthy weight	27.5 (21–34)	28.9	26.1	

a Chi-square test.

b Fisher's exact test.

c n = 178.

Bold values: p < 0.05.

**Table 2:** Women's disease characteristics according to the two groups of depression symptoms. Oncological Research Center (CEPON). Florianópolis, state of Santa Catarina, Brazil, 2014/2015. (n = 181)

Variable		Total	Absence of depression symptoms	Presence of depression symptoms	p
		% (95%CI)	%	%	
Presence of other disease					**0.014**
	Yes	49.2 (42–56)	40.2	58.4	
	No	50.8 (43–58)	59.8	41.6	
Type of surgery*					**0.002**
	Radical mastectomy	61.4 (54–68)	50.0	72.9	
	Breast-conserving surgery	38.6 (31–45)	50.0	27.1	
Breast reconstruction*					0.895
	No	74.9 (68–81)	74.4	75.3	
	Yes	25.1 (18–32)	25.6	24.7	
Presence of lymphedema*					**0.018**
	Yes	45.0 (37–52)	36.0	54.1	
	No	55.0 (47–62)	64.0	45.9	
Physical therapy					0.065
	Yes	57.5 (50–65)1	64.1	50.6	
	No	42.5 (35–50)	35.9	49.4	
Treatment stage					0.690
	After treatment	23.8 (17–30)	25.0	22.5	
	In treatment	76.2 (69–82)	75.0	77.5	

* Analyzed only between the women who have undergone the surgery (n = 171). Chi-Square test.

Bold values: p < 0.05.

The most frequent forms of treatment for the women in this study were chemotherapy (41.5%), radiotherapy (26.2%), and hormone therapy (32.3%) (data not shown). At the time of the study, most women had already finished the treatment 12 months before. Having other diseases beyond BC was associated with the presence of depression symptoms. The most frequent comorbidity was cardiovascular disease (31.5%), followed by metabolic disease (17.1%), and osteoarticular disease (13.8%) (data not shown). Surgery type was associated with the presence of depression symptoms, especially radical mastectomy. The presence of lymphedema was also associated with depression symptoms.

Most women showed high self-esteem, and those with medium or low self-esteem showed more depression symptoms. Significant differences between the groups were identified regarding body image: women with depression symptoms had worse body image on all six of the questionnaire scales (Table 3).

**Table 3:** Women's self-esteem and body image according to the two groups of depression symptoms. Oncological Research Center (CEPON). Florianópolis, state of Santa Catarina, Brazil, 2014/2015. (n = 181)

Variable		Total	Absence of depression symptoms	Presence of depression symptoms	p
		% (95%CI)	%	%	
Self-esteem^a^					**< 0.001**
	High	70.2 (63–76)	85.9	53.9	
	Medium	24.9 (18–31)	12.0	38.2	
	Low	5.0 (2–8)	2.2	7.9	
Body image^b^,^c^		Md (IQR)	Md (IQR)	Md (IQR)	
	Vulnerability	19.0 (12)	15.0 (11.5)	24.0 (12.0)	**< 0.001**
	Body stigma	25.0 (17)	20.0 (12.0)	29.0 (17.0)	**< 0.001**
	Limitations	14.0 (8)	11.0 (6.0)	18.0 (8.0)	**< 0.001**
	Body concerns	14.0 (9)	12.0 (9.5)	15.0 (10.0)	**< 0,001**
	Transparency	9.5 (9)	7.0 (5.0)	14.0 (11.0)	**< 0.001**
	Arm concerns	7.0 (7)	5.0 (3.0)	10.0 (5.0)	**< 0.001**

Md: median; IQR: interquartile range

a Fisher's exact test.

b Only in women who have completed the questionnaire (n = 168).

c Mann Whitney *U* test.

Bold values: p < 0.05.

Table 4 shows that, when adjusted for general characteristics (block 1), age and education attainment level were significant: younger women (40–59 years old) had a 55.7% prevalence ratio of depression symptoms and women with only an elementary school education had a 50.8% prevalence ratio of depression symptoms. Similarly, the analysis adjusted for disease characteristics (block 2) shows that women with other diseases had a 38.3% prevalence ratio of depression symptoms, and those who had undergone surgery of radical mastectomy had a 63.0% prevalence ratio of depression symptoms.

**Table 4:** Prevalence ratios for the presence of depression symptoms in participating women. Oncological Research Center (CEPON). Florianópolis, state of Santa Catarina, Brazil, 2014/2015. (n = 181)

Variable		PR Adjusted for each block	p	PR Adjusted for all variables	p
		(95%CI)		(95%CI)	
Block 1					
Age (years old)			**0.018**		0.669
	40–60	1.557 (1.079–2.248)		1.074 (0.776–1.486)	
	61–80	1.00		1.00	
Educational attainment			**0.010**		**0.030**
	Basic school	1.508 (1.101–2.067)		1.371 (1.031–1.823)	
	High school and undergraduate degree	1.00		1.00	
Present job			0.781		
	Home	0.803 (0.408–1.557)		-	
	Unemployed/Retired/Expertise	0.748 (0.325–1.721)		-	
	One or two jobs	1.00		-	
Marital status			0.480		
	With partner	1.115 (0.825–1.507)		-	
	Without partner	1.00		-	
Economic level			0.402		
	Low level (D+E)	1.473 (0.826–2.625)		-	
	Medium level (C)	1.403 (0.828–1.507)		-	
	High level (A+B)	1.00		-	
Weight status^a^			0.742		
	Overweight	1.059 (0.753–1.490)		-	
	Healthy weight	1.00		-	
Block 2					
Other disease			**0.039**		0.162
	Yes	1.383 (1.016–1.882)		1.215 (0.925–1.597)	
	No	1.00		1.00	
Treatment stage			0.254		
	After treatment	0.822 (0.586–1.151)		-	
	In treatment	1.00		-	
Type of surgery^b^			**0.010**		0.147
	Radical mastecomy	1.630 (1.127–2.359)		1.256 (0.923-1.710)	
	Breast-conserving surgery	1.00		1.00	
Breast reconstruction^b^			0.473		
	No	1.132 (0.807–1.587)		-	
	Yes	1.00		-	
Presence of lymphedema^b^			0.203		
	Yes	1.213 (0.885–1.662)		-	
	No	1.00		-	
Physical therapy			0.472		
	Yes	0.894 (0.659–1.213)		-	
	No	1.00		-	
Block 3					
Self-esteem			0.274		0.097
	High	1.233 (0.734–2.070)		1.318 (0.763–2.278)	
	Medium	1.408 (0.896–2.212)		1.569 (0.973–2.531)	
	Low	1.00		1.00	
Body image^c^					
	Vulnerability	1.007 (0.988–1.026)	0.461		
	Body stigma	0.989 (0.975–1.003)	0.125	0.988 (0.975–1.001)	0.062
	Limitations	1.044 (1.009–1.080)	**0.013**	1.050 (1.016-1.086)	**0.004**
	Body concerns	1.007 (0.984–1.031)	0.534	-	
	Transparency	1.049 (1.022–1.078)	**< 0.001**	1.046 (1.021–1.071)	**< 0.001**
	Arm concerns	1.062 (1.015–1.112)	**0.010**	1.067 (1.022–1.113)	**0.003**

a n = 89

b n = 171

c n = 168

Bold values: p < 0.05.

After adjusting by all variables, educational attainment was significant. Women who studied until elementary school had a prevalence ratio of 37.1% compared to those who studied until high school or further. For the analysis of body image, limitation, transparency, and body concerns, the scales remained significant, meaning that adding one more point in the score could increase the prevalence ratio of depression symptoms by 5.0%, 4.6%, and 6.7%, respectively.

## DISCUSSION

Despite technological progress in medicine and increases in survival rates, cancer is still associated with a risk of death. Cancer diagnosis causes psychological issues such as fear, sadness, and depression^3^. An international study determined that, among the various types of cancer, women diagnosed with BC were the ones that presented more depressive disorders^7^. Therefore, this study analyzed the factors associated with the presence of depression symptoms in women after a diagnosis of BC. Almost half of the subjects analyzed presented depression symptoms. Women that presented depressive symptoms also presented low and medium self-esteem, were younger, were diagnosed with other diseases, had received a radical mastectomy, and presented lymphedema. A higher prevalence of depression symptoms was also related to less education and lower scores on the body image, in the scales of limitations, transparency and arm concerns.

Women with higher self-confidence, higher emotional stability, positive thoughts, and higher self-esteem have a higher psychological well-being when battling BC^3^. This notion is consistent with our results as women with better self-esteem displayed less depression symptoms. Since self-esteem is influenced by health perception, religious beliefs, family support, and economic levels, it is important to note that most of the women in this study were classified as having lower income, less education, and were diagnosed with other diseases; therefore, it is possible that these characteristics affected their self-esteem and worsened their depression symptoms.

Low self-esteem is related to the incapacity of facing challenges^21^. Therefore, the experience and consequences of BC treatment likely causes deterioration in women's quality of life^5^. Cancer thus becomes a life challenge. In these situations, different reactions are observed in the diagnosis of the disease. Some of them remain positive, with thoughts of hope, while others may deny the disease, isolate themselves from family, and display personal disappointment^22^. These feelings can cause low self-esteem, which increases depressive symptoms.

Similarly, women's body image undergoes physical and psychological changes during BC^2^, and these modifications can be related to depressive disorders^6^. Our results are consistent with this affirmation as the women that presented depressive symptoms also had the worst scores on all body image scales. A previous study showed that only 27% of women after BC surgery felt comfortable with their own body^2^. This dissatisfaction and discomfort with their own body after surgery, along with the treatment procedures, results in a worse self-perception of their body and leads to depression symptoms, denial, isolation, sadness, and a pessimistic view of the future^3^ ^,^ ^5^.

The regression analysis showed that only the scales limitation, transparency, and arm concerns were statistically significant after the adjusted analysis. More specifically, the limitation scale addressed women's sense of competence and ability in relation with their own body. Moreover, transparency addressed the changes in body appearance in relation to the cancer^15^. Physical changes resulting from radical mastectomy have possibly lead these women toward difficult adaptations to the functional activities of daily living and increased the dissatisfaction with their own bodies^2^. Changes in body image also worsen women's sexuality, creating a fear of rejection by their partners and increasing feelings of sadness and isolation^1^ ^,^ ^3^. In addition, the removal of the breasts can complicate women's sexual identity and feelings of motherhood, which could deteriorate competence and family relationships^3^.

Some psychological effects from BC surgery were highlighted in this study. A high association between radical mastectomy and depression symptoms was found. Women who went through radical mastectomy had a 63% prevalence rate of depression symptoms when compared to ones that underwent conservatory surgery. Previous studies showed similar results in Brazilian^23^ and Polish^4^ women.

Lymphedema is a possible consequence of BC surgery that affects nearly 30% of women after the surgery and is characterized as a chronic condition^24^. In this study, lymphedema was associated to depression symptoms, considering that 54.1% of the women with depression symptoms were related to lymphedema. This indicated that learning to live with lymphedema could be a causal factor to frustration, sadness, and depression, because activities of daily living become more difficult and require more time, dedication, and care^25^.

In addition, lower scores regarding arm concerns indicated a higher prevalence of depression symptoms. Concerns resulting from arm symptoms are related to the presence of lymphedema^26^ and worse body image, which could cause depression symptoms^27^. The presence of lymphedema alters women's routine activities, making it difficult to dress, for example. Usually, these women begin to wear only loose-fitting, long-sleeve clothes that can hide the volume of the compromised arm. This situation results in negative well-being and emotional aspects, which can lead to depressive symptoms.

Presence of other diseases besides BC was also associated with depression symptoms. The results showed that cardiovascular diseases were the type of disease besides cancer that was responsible for most of the diseases in the women in this study. According to Armenian et al.^28^, the BC women can show lower survival rates because of cardiovascular outcomes, such as late effects of therapy. It is well known that women who survive cancer present more risk factors related to cardiovascular diseases than people without cancer do, except for those who are smokers^29^, and this other diagnosis along with cancer can lead to more depressive symptoms, as these women present lower survival rates and have more fear about the future. This data should be carefully analyzed because the time when the other diseases were diagnosed was not controlled. Therefore, one cannot know if it happened prior, during, or after the BC treatment.

Age was also an important factor as women aged 40–60 years had more depressive symptoms than women aged 61–80 years. In the block for adjusted regression analysis by general characteristics, the age group also showed significance, with a 55% prevalence ratio of depression symptoms in younger women. Younger women are typically more concerned with infertility because of chemotherapy treatment since it reduces the production of female hormones, shortens the period before menopause, and can cause infertility^29^. Furthermore, body image tends to be more important in younger women^30^; therefore, such women have more body afflictions and fear of disease recurrence, social isolation, and family and marital problems^30^. This information could assist interdisciplinary care guidance, suggesting that psychological support should be included early in treatment.

A lower educational attainment was also linked with depression symptoms: women with a basic education presented a 37% prevalence ratio of depression symptoms when compared with women with at least a high school degree. Lack of knowledge about the disease can complicate women's emotions; therefore, if they do not have access to information about treatment and survival rate, the main thought becomes one's end of life^3^, which can cause more depressive symptoms. Most of the women in this study were retired, unemployed, waiting for health retirement diagnostic, and had low income. These factors could influence psychological aspects considering that women begin to worry about their future, returning to work and financial situation after treatment.

Some limitations must be addressed: the use of a questionnaire as a data collection tool could be a limiting factor for the depression diagnosis. Additionally, the use of antidepressant medication could have altered women's perceptions of their depressive symptoms. Also, the previous depression diagnostic criteria were not controlled. Another limitation of the study may be the fact that most women were still in clinical BC treatment during the study period. However, this variable was analyzed as covariate and presented no significant results in the analysis.

Despite the limitations in relation to the sample size, as well as the selection of the sample, which in turn was for convenience, our findings are essential for studying BC in the national context. These results can subsidize new interventions directed especially to the less favored populations, those of lower educational level. In developing countries such as Brazil, the access to information by these women of less education is more difficult and, because of that, this type of study can help professionals to turn their eyes to this problem.

## CONCLUSIONS

After being diagnosed with BC, women who are younger, are less educated, have been diagnosed with other diseases, had radical mastectomy, have lymphedema, have low to average self-esteem, and have low body image scores presented more depressive symptoms. Therefore, it is important that healthcare professionals are aware of these relationships and try to detect depression symptoms earlier and improve the care they provide these women.

However, after an adjusted Poisson regression analysis by all variables included in the study, only the scales educational attainment, body image limitation, transparency and arm concerns resulted in a higher prevalence of depression symptoms. Therefore, women require more education about and multidisciplinary attention towards the disease. In turn, women who display poor self-perception and negative body judgment during BC treatment should receive support as these factors may increase depression symptoms.
